# 全孔道（port-only）人工气胸下机器人辅助肺叶切除术

**DOI:** 10.3779/j.issn.1009-3419.2020.01.08

**Published:** 2020-01-20

**Authors:** 连民 张, 晓亮 赵, 峰 徐, 钰 张, 强 张, 健 尤

**Affiliations:** 300060 天津，天津医科大学肿瘤医院，肿瘤研究所，国家癌症临床研究中心，肿瘤防治重点实验室，天津市肿瘤临床研究中心 Department of Lung Cancer, Tianjin Medical University Cancer Institute and Hospital, National Clinical Research Center for Cancer, Key Laboratory of Cancer Prevention and Therapy, Tianjin's Clinical Research Center for Cancer, Tianjin Lung Cancer Center, Tianjin 300060, China

**Keywords:** 肺癌, 机器人辅助胸外科, 全孔道, 优化, Lung cancer, Robot-assisted thoracic surgery, Port-only, Optimization

## Abstract

**背景与目的:**

达芬奇机器人手术操作系统目前在胸外科得到了广泛的应用，国内不同中心采用的建立通道方式及操作流程，不同辅助操作孔多采用小切口的方式。

**方法:**

基于大量临床实践的基础上，我们在实践中总结国内、外经验，并结合国人体型特点，对机器人辅助肺叶切除术的切口及操作流程进行了改进并实践出一套更加简化易行的手术方法。

**结果:**

全孔道（port-only）人工气胸下机器人辅助肺叶切除术在术中解剖安全性，止血效果和伤口美观性方面，都有进一步的提升。

**结论:**

本研究对全孔道人工气胸机器人辅助肺叶切除的流程加以优化，以期为肺癌患者更好地服务。

## Abstract (en)

**Background and objective:**

Da Vinci robotic system is currently widely used in thoracic surgery. The ports employment and procedures vary in different medical center in China. Usually, a small incision was used for assistant.

**Methods:**

Based on clinical practice, we summarized domestic and foreign experience, combined with the characteristics of the Chinese body anatomy, employ portal technique and artificial pneumothorax, summarized a set of simplified and easier surgical method.

**Results:**

Port-only artificial pneumothorax robot-assisted lobectomy has further improvement in anatomical safety, hemostatic effect and aesthetic appearance of the wound.

**Conclusion:**

This study optimizes the procedure of port-only artificial pneumothorax robot-assisted lobectomy in order to serve lung cancer patients better.

达芬奇机器人手术系统的设计理念是通过微创的方法实施复杂的外科手术，达芬奇系统主要由三部分组成：外科医师控制台、床旁机械臂系统及高清成像系统。主刀医师坐在控制台中，位于手术室无菌区外，甚至可以远程操控来完成手术操作。床旁机械臂系统是外科手术机器人的主要操作部件，其主要功能是为器械臂和摄像臂提供支撑，助手医师在无菌区内的床旁机械臂系统边进行辅助操作，负责更换器械及应用切割闭合器等，协助主刀医师完成手术。随着机器人系统不断升级，助手的作用不断弱化。胸腔天然的骨性结构使机器人打孔位置有所限制，但天然的操作空间也使机器人手术在胸外科得到了广泛的应用。我们在现有常规机器人手术操作流程的前提下，采用可视化打孔方式并配合人工气胸的方法进一步优化机器人肺癌切除的流程，以期为更多的肺癌患者服务。

## 1 资料与方法

### 1.1 一般资料

我院自2016年初引进达芬奇机器人手术系统以来，应用全孔道人工气胸操作方式，单一术者共完成肺外科手术120余例。适应症与常规胸腔镜手术相同，常规术前检查，对临床Ⅰ期-IIIa期可手术患者行机器人辅助肺癌根治术，手术方式包括肺叶切除，肺段切除，袖状切除及系统性淋巴结清扫等。

### 1.2 体位和麻醉

全身麻醉，气管内双腔插管，90度侧卧折刀位，胸下垫塑形垫固定体位。适当折刀位避免镜头机械臂对髋骨的压迫，增大肋间隙（图 1A，图 1B）。手术野常规消毒铺巾，术前打孔常规罗哌卡因肋间及椎旁阻滞，减少穿刺器对肋间神经的压迫损伤，减轻术后伤口疼痛。机器臂系统放置在患者的头侧，助手始终站在患者腹侧（图 1C）。

**1:**
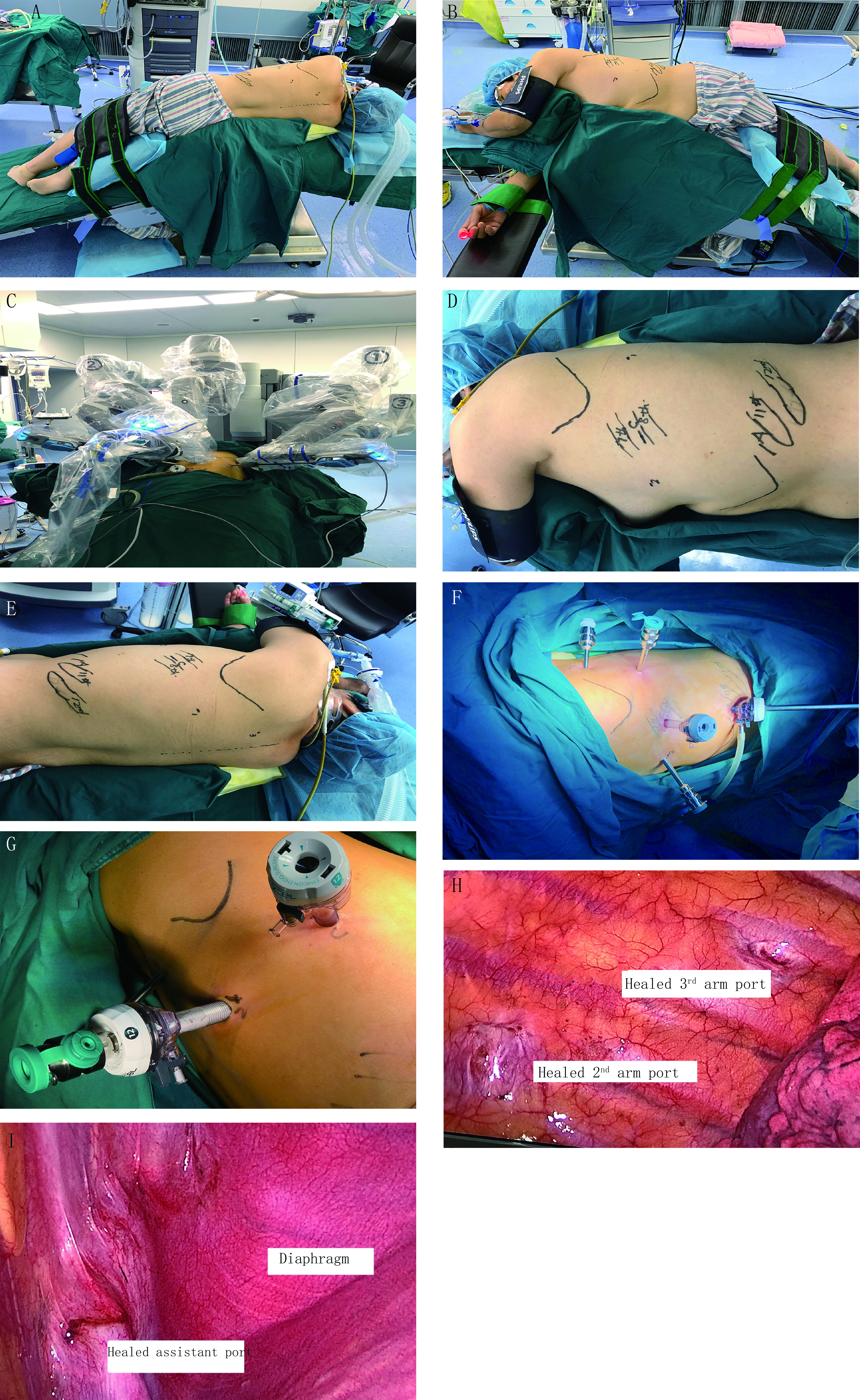
病例图片。A：体位背侧视图；B：体位腹侧视图；C：机械臂摆放视图；D：术前规划孔道（腹侧视图）；E：术前规划孔道（背侧视图）；F：孔道建立后视图；G：右肺中叶手术12 mm套8 mm孔道；H：术后1年胸内背部孔道愈合视图；I：术后1年辅助操作孔愈合视图。 Case pictures. A: dorsal view; B: ventral view; C: robotic arm placement; D: plan out the assistant port camera port and instrument arm port prior to incision of the patient (ventral view); E: plan out the assistant port camera port and instrument arm port prior to incision of the patient (dorsal view); F: view of employed ports; G: diagram of insert 8mm robotic trocar in 12 mm trocar; H: the view of healed port one year after surgery; I: the view of healed assistant port one year after surgery.

### 1.3 切口选择

机器人系统为DaVinci Si system（Intuitive Surgical, Sunnyvale, CA）。所有操作孔道建立均可视化，电刀仅切开皮肤及皮下组织，并不切开肌层，而由穿刺器钝性分离进入胸腔。首先建立镜孔（12mm）选在腋中线7或8肋间，机器人镜头30°向上，检查胸腔有无广泛粘连，于镜孔建立人工气胸，压力4 mm-6 mm汞柱，流量12 L/min-15 L/min；然后可视下建立辅助操作孔（12 mm），通常位于10肋间（11肋上缘），如患者先天11肋过短，或者下肺切除时可适当上移至9肋间，主要协助压肺、放置可调节吸引器和切割缝合器以及拿取标本及淋巴结；机器人镜头和气胸管移至辅助操作孔，在全可视下建立其他操作孔，第一臂操作孔（8 mm）和第二臂操作孔（8 mm），根据患者体型及左右肺不同可选腋前线稍微偏前和肩胛下角线稍偏后与镜孔同一肋间或上下移1个肋间，原则上确保后部的操作孔位置低于斜裂；第三臂操作孔（5 mm）位于脊柱旁线，第4或5肋间，根据不同胸廓形状调整（图 1D，图 1E，图 1F）。切口位置并非绝对，仍采取个体化原则，根据患者体型及左右肺的不同做调整。常用器械包括8 mm马里兰钳（Maryland Bipolar Forceps）、8 mm无损伤抓钳（Cardiere Forceps）、5 mm胸科抓钳（Thoracic Grasper）及针持等，并且应用自制小纱布卷推压肺组织暴露解剖部位，极大的减少正常肺组织牵拉钳夹。术毕，适当延长辅助操作孔取标本，因为辅助孔位于肋弓膈肌反折处，组织间隙较大，易于取出标本。打孔流程视频见http://dx.doi.org/10.3779/j.issn.1009-3419.2020.01.12。

### 1.4 手术方式及流程优化

#### 1.4.1 右肺上叶

推荐应用后入路支气管优先处理原则。首先马里兰钳配合无损伤抓钳先解剖后部斜裂，游离后升支动脉，三臂夹持纱布卷压肺向前，解离上叶支气管后部和下缘，同时清扫隆突下淋巴结，然后闭合器切割分离后部肺裂，闭合切断后升支动脉。三臂压上肺向下，解剖支气管上缘，同时清扫上纵隔淋巴结，三臂拉肺向后部，暴露前肺门，马里兰钳锐性配合钝性分离上、中叶间间隙，游离上肺静脉；三臂牵起上叶后部，辅助操作孔进入切割缝合器，闭合切断上叶支气管，切断气管后，三臂牵拉支气管断端，分离闭合切断尖前动脉干，然后闭合器切断上肺静脉，最后闭合器处理水平裂，此流程最大限度的减少水平裂的影响，极大的减少肺脏的翻动，易于操作。

#### 1.4.2 右肺中叶

因为解剖关系，二臂操作孔可以12 mm戳卡套入8 mm机器人戳卡（图 1G）。首先锐性解离打开前部斜裂，游离暴露A4，辅助操作孔进入切割缝合器切断A4，马里兰配合无损伤抓钳游离中叶静脉，二臂操作孔进入切割缝合器闭合切断中叶静脉，再解离右肺中叶支气管并清扫气管旁淋巴结，二臂操作孔进切割缝合器切断中叶支气管，同样二臂进切割缝合器处理A5动脉和发育不全的水平裂。机器人右肺中叶视频见http://dx.doi.org/10.3779/j.issn.1009-3419.2020.01.13。

#### 1.4.3 下叶切除

左肺下叶处理方式与右肺下叶处理方式近似，在此一并加以说明，首先处理叶间裂，锐性解离叶间裂，马里兰钳配合切割闭合器打开叶间裂，再分离下叶背段及基底段动脉，其次处理下叶支气管，并清扫支气管周围淋巴结，最后分离下肺韧带，解剖出下肺静脉，并离断，下叶切除较上叶切除更为简单易行；如果肺裂发育极差，也可以首先处理下肺韧带，切断下肺静脉，然后解离下叶支气管并切断，最后处理下肺动脉和不全的叶裂，所有切割缝合器均从辅助操作孔进入。

#### 1.4.4 左肺上叶

左肺上叶动脉分支多，变异大，此处仅讨论常见血管分支情况，同样遵循从后向前的顺序，首先处理叶间裂，锐性解离叶间裂，能量器械配合切割闭合器打开叶间裂，游离并切断舌段动脉，向上解离，游离并切断A1+2^c^，然后处理A1+2^a+b^及A3，接着处理上叶支气管，游离上叶气管并清扫周围淋巴结，最后处理上肺静脉。处理支气管和左肺动脉第一支的顺序可以根据动脉解剖位置或者支气管根部淋巴结情况互换，所有切割闭合器均从辅助孔进入操作。

#### 1.4.5 系统性淋巴结清扫

系统清扫较常规胸腔镜更为容易，马里兰钳配合无损伤心包抓钳，5 mm抓钳抓持小纱布卷推拉组织暴露，几乎不需吸引器的暴露及吸引，无论对于隆突下淋巴结还是上纵隔淋巴结均可整块切除，而且由于马里兰钳双极电凝止血彻底，能充分保证术野干净清晰。

### 1.5 器械使用技巧

#### 1.5.1 体位技巧

身下垫塑形垫，90度卧位轻度前倾，适当折刀，手术床于剑突上水平，如行上叶切除，适当头部抬高，如中下肺叶切除，保持胸部水平，利于打开肺叶自然暴露。

#### 1.5.2 肺叶切除及淋巴结清扫流程技巧

进胸后先从叶裂处解剖开始，减少肺叶翻动，叶裂薄弱处解剖出肺动脉，同时等候肺叶残气排出和吸收。解剖上肺叶上部肺门时，一并清扫上纵隔淋巴结，解剖后部肺门时，同时清扫隆突下淋巴结，目的同样为了减少翻动肺叶。

#### 1.5.3 马里兰钳操作技巧

利用马里兰钳的双极电凝，可以极其安全在大血管周围解剖，不必担心周围副损伤。以马里兰钳夹组织，电凝后以垂直于切割方向快速拉扯，可以安全分离组织。

#### 1.5.4 血管处理技巧

多数血管可以马里兰钳分离后，通过切割缝合器切断。个别较细或者进切割缝合器角度不顺畅的血管，可以马里兰钳和心包抓钳配合结扎后切断，内镜下的锁扣夹也是可以选择的处理方式。

#### 1.5.5 叶裂处理技巧

相对发育不全的肺裂，可以从叶裂薄弱处开始解剖，以马里兰钳分离至正确血管鞘外间隙后，适当电凝扩大，完全通过后，以切割缝合器切断。通常先处理后部肺裂。融合粘连难以过切割缝合器分离的肺裂可以通过马里兰钳配合心包抓钳缝合后切断。

## 2 结果

全组患者无围术期死亡。1例因广泛致密胸膜粘连，另1例因肺动脉周围广泛陈旧结核性淋巴结粘连镜下难以游离，中转开胸手术。全组手术学习曲线如表 1。

**1:** 全孔道人工气胸下肺叶切除术学习曲线 The learning curve of Port-only robot-assisted lobectomy

	First 50 cases	Subsequent 70 cases
Port employment & docking time (min)	36±13	15±9
Operative time (min)	145±23	95±19
Serious complications	0	0
Postoperative morbidity	0	0
Bleeding volume (mL)	40±10	25±12

## 3 讨论

目前，国内外很多学者认为机器人辅助下胸腔镜手术（robot-assisted thoracoscopic surgery, RATS）相较于传统胸腔镜手术有着更高的操控性、精确性，特别是稳定性^[1-5]^；尤其对于复杂手术及重建手术^[6]^，优势更为明显；创新的内转腕系统和可多维度自由活动的机械臂可使镜下手术器械完全重现人手动作从而达到手眼协调；更为重要的是，操作系统可以过滤主刀医生手部颤抖对手术所造成的不利影响；并且最大的创新性是使远程操作成为可能。在胸外科领域，机器人手术系统已经得到了广泛的应用^[7-9]^，目前国内达芬奇机器人系统已逾百台。

首先，在可行性方面，有学者报道了比较常规开胸肺叶切除和四臂法机器人肺叶切除手术^[10]^，术后住院时间方面机器人组较短，但是手术时间对比开胸组要长，可随着学习曲线的结束，手术时间明显缩短，手术过程更为流畅^[11]^，这与我们的实践完全一致。我们认为成熟的机器人手术在纵隔淋巴结清扫、肺门及肺血管的解剖方面有明显优势^[12]^，尤其对于重建手术，机器人操作系统优势更为明显，并且自制小纱布卷推肺，减少肺的牵拉，同时保证术野干净，全打孔机器人操作流程建立了大量病例基础上，可以明显缩短学习曲线。另外配合低压力人工气胸，可以降低膈肌，使辅助操作孔可以低至10肋间肋弓处，这样可使进闭合器方向平行于纵隔，顺畅处理绝大多数的肺血管和所有支气管。解剖分离组织多应用马里兰钳的双极电凝功能，只对钳夹的组织电凝切割，产烟较少，止血彻底，更适合全孔道人工气胸下的解离操作。

在安全性方面，有学者系统性比较了微创肺叶切除手术和常规开胸肺叶切除手术用于治疗早期肺癌的短期并发症和长期生存率，微创肺叶切除手术被认为可使患者获得显著的生存获益，同时也表明微创手术对患者免疫抑制影响较小^[13]^。同时也有学者对325例行机器人肺叶切除治疗的可手术非小细胞肺癌患者进行随访，这其中76%为Ⅰ期肺癌，18%为Ⅱ期肺癌，6%为Ⅲ期肺癌；中位随访期达到27个月，5年生存率高达80%。因此，目前有限的随访数据表明了机器人肺叶切除手术生存率是可以接受的^[14, 15]^。在本研究中，行全孔道机器人辅助手术患者尚无复发转移，但仍需更长的随访时间，进一步评估该手术方式对肺癌患者预后的影响。

生活质量方面，我们的研究发现全孔道机器人辅助手术较常规胸腔镜手术及开胸手术疼痛明显降低^[16]^，止疼药物用量明显减少，尤其配合术前罗哌卡因进行椎旁和肋间神经阻滞，明显降低的疼痛相关并发症，更利于术后排痰，全孔道下机器人手术，术后切口更为美观，肌肉损伤小，不影响临近器官功能，并且全打孔，术后胸腔内粘连更轻，利于二次手术的进行。本研究中有1例行机器人辅助右肺中叶肺癌手术的患者，1年后同侧其他肺叶再次行胸腔镜手术，可见全孔道机器人手术后胸腔内无任何粘连（图 1H，图 1I）。

机器人手术带给术者的那种超越人手的灵活性、体力和精力的节约是电视胸腔镜手术无法比拟的，但是机械臂缺乏力反馈，无法代替人手的触觉感受，经验丰富的机器人操作医生可以凭借超级放大的视野，在一定程度上模拟感受器械操作力度，短期内机器人手术仍然无法完全代替传统的微创手术和开放手术，机器人毕竟只是人手的延伸。本文总结的全孔道人工气胸下机器人手术操作流程和方法是建立在大量胸腔镜和开放手术基础上的改进，目的是通过最小的创伤达到根治的目标，造福更多肺癌患者。
